# Co-expression, purification, and characterization of an acidophilic and n-hexane-tolerant lipase with its foldase from *Burkholderia gladioli* Bsp-1

**DOI:** 10.1007/s00253-026-13788-z

**Published:** 2026-03-28

**Authors:** Jing Zhu, Xiaoqiong Zuo, Lanqiu Mai, Yan Qin, Liang Xian, Yi Li, Qingyan Wang

**Affiliations:** 1 College of Food and Quality Engineering, Nanning University, Nanning, 530200 People’s Republic of China; 2 State Key Laboratory of Non-Food Biomass Energy Technology, Guangxi Academy of Sciences, Nanning, 530007 People’s Republic of China

**Keywords:** *Burkholderia gladioli* Bsp-1, Acidophilic, Lipase, Co-expression, Characterization, Foldase

## Abstract

**Abstract:**

Lipases are versatile biocatalysts widely applied in hydrolysis and synthesis reactions, yet bacterial acidophilic and solvent-tolerant lipases remain poorly characterized. In this study, an acid- and organic solvent-resistant lipase (LipC) and its cognate foldase (LifB) from *Burkholderia gladioli* Bsp-1 were cloned and heterologously expressed in *Escherichia coli*. Soluble and catalytically active LipC was obtained only upon co-expression with LifB, demonstrating a strict foldase-dependent folding requirement. Phylogenetic analysis classified LipC as a LipA-type bacterial lipase. Biochemical characterization revealed that LipC exhibited maximal activity at pH 3.5 and showed remarkable stability under acidic conditions, retaining more than 70% of its activity after prolonged incubation at pH 4.0. The enzyme displayed optimal activity at 55 ℃ and maintained moderate thermal stability. Notably, LipC retained high activity in nonpolar organic solvents, with significant activation observed in n-hexane and cyclohexane. Substrate specificity and kinetic analysis indicated a preference for medium-chain fatty acid esters, with the highest catalytic efficiency toward p-nitrophenyl caprate (C10). Collectively, these results identify LipC as a foldase-dependent bacterial lipase combining acidophilic behavior and solvent tolerance, expanding the current understanding of bacterial lipases and highlighting their potential relevance for biocatalysis under acidic and nonaqueous conditions.

**Key points:**

• *LipC is a foldase-dependent bacterial lipase requiring LifB for functional expression*.

• *LipC exhibits strong acidophilicity with optimal activity at pH 3.5*.

• *LipC is highly activated by nonpolar solvents such as n-hexane and cyclohexane*.

**Supplementary Information:**

The online version contains supplementary material available at 10.1007/s00253-026-13788-z.

## Introduction

Lipases (EC 3.1.1.3) are α/β-hydrolase enzymes that catalyze the hydrolysis of ester bonds and, under low-water conditions, mediate esterification and transesterification reactions (Castilla et al. 2022). Owing to their conserved catalytic triad and structural fold, lipases display broad catalytic versatility and are widely applied in food processing, lipid modification, and fine chemical synthesis (Akram et al. 2023; Castilla et al. 2022). Among them, microbial lipases are of particular interest because of their functional diversity, stable supply, and amenability to heterologous expression and molecular engineering (Akram et al. 2023).

Bacterial lipases, particularly those from Gram-negative genera such as *Pseudomonas*, *Burkholderia*, and *Serratia*, represent a major source of industrially relevant enzymes due to their structural diversity, broad temperature adaptability, and amenability to protein engineering. Representative examples have been extensively reported in *Pseudomonas* (Rios et al. 2018; Yu et al. 2022), *Burkholderia* (Shu et al. 2016), and *Serratia* species (Jiang et al. 2016; Mohammadi et al. 2016). However, heterologous expression of these lipases in *Escherichia coli* often remains challenging, as many enzymes accumulate as insoluble or inactive proteins due to the complexity of lipase folding (Guan et al. 2020; Jiang et al. 2016; Mohammadi et al. 2016). A subset of bacterial lipases even requires dedicated lipase-specific foldases (Lim, LipB, or Lif) for productive folding and activation, as first demonstrated in *Pseudomonas* systems and later extended to *Burkholderia species* (Hausmann 2009; Hobson et al. 1993; Madan et al. 2010; Pauwels 2008; Zhang et al. 2021). Structural studies further revealed that these foldases act as steric chaperones that stabilize productive folding intermediates of lipases (Hobson et al. 1995; Knapp et al. 2016). Nevertheless, foldase dependence cannot be reliably inferred from sequence information alone and must be experimentally verified.

Acidophilic lipases have been studied predominantly in filamentous fungi and yeasts, particularly *Aspergillus* and *Candida* species, which typically exhibit pH optima between 3.0 and 5.0 and show strong stability under acidic conditions (ANTONIA 2003; Mtibaa et al. 2002; Shi et al. 2014; Zhu 2007; Zhu et al. 2019b). In contrast, bacterial acidophilic lipases remain relatively uncommon, as most characterized bacterial lipases display neutral or alkaline pH optima. Nevertheless, several notable examples have been reported, including the acidic lipase Lip I.3 from a *Pseudomonas fluorescens*-like strain (Panizza et al. 2013), an acidic lipase from Pseudomonas gessardii (Kandasamy et al. 2010), and extremely acidic lipases produced by Bacillus species (Saranya et al. 2014). Despite these reports, bacterial lipases with pronounced activity under strongly acidic conditions are still comparatively limited.

Lipases have also attracted considerable interest for catalysis in organic or low-water media, where nonaqueous systems improve substrate solubility and favor synthesis over hydrolysis (Jian et al. 2022; Koskinen and Klibanov 1996; Salihu and Alam 2015). Although fungal and immobilized lipases have been widely exploited for such applications (Graber et al. 2008; Löfgren et al. 2019; Qian et al. 2025), naturally occurring bacterial lipases that combine acidophilicity with solvent tolerance remain uncommon.

*Burkholderia gladioli* Bsp-1 was previously isolated and identified in our laboratory as a lipase-producing strain (Zhu et al. 2019a). In our earlier work, we characterized an alkaline extracellular lipase from this strain; however, genome analysis further revealed the presence of additional putative lipase genes whose functional roles had not been explored. Among them, LipC was annotated as a putative LipA-type bacterial lipase based on sequence homology (Arpigny and Jaeger 1999; Ding et al. 2019), but its foldase requirement and biochemical properties have not been established.

In the present study, LipC and its cognate foldase LifB were co-expressed in *E. coli*, and the resulting enzyme was characterized with respect to folding dependence, pH and temperature profiles, solvent tolerance, substrate specificity, and catalytic efficiency, in order to position LipC within the functional landscape of bacterial lipases operating under acidic and nonaqueous conditions.

## Materials and methods

### Bacterial strains, plasmids, and materials

*Burkholderia gladioli* Bsp-1 was screened and preserved in our laboratory and has been deposited in the China General Microbiological Culture Collection Center (CGMCC No. 10533). *Escherichia coli* JM109 was used for plasmid propagation, while *E. coli* BL21(DE3) and *E. coli* Origami 2(DE3) (Invitrogen, CA, USA) were used as expression hosts. The expression vectors pET-22b(+) and pETDuet-1 were obtained from Invitrogen (CA, USA).

Restriction endonucleases, the In-Fusion HD Cloning Plus Kit, and DNA polymerases were purchased from Takara Biotechnology (Dalian, China). The OMEGA Gel Extraction Kit was obtained from Omega Bio-tek (USA). All other reagents were of analytical grade unless otherwise stated.

### Media composition

Luria–Bertani (LB) medium consisted of tryptone (10 g/L), yeast extract (5 g/L), and NaCl (10 g/L), with the pH adjusted to 7.0.

The auto-induction medium contained peptone (10 g/L), yeast extract (5 g/L), glycerol (5 g/L), glucose (0.5 g/L), lactose (5 g/L), Na_2_HPO_4_·12H_2_O (18 g/L), KH_2_PO_4_ (7.0 g/L), NH_4_Cl (2.5 g/L), Na_2_SO_4_ (0.7 g/L), MgSO_4_·7H_2_O (0.5 g/L), and ampicillin (100 μg/mL).

### Phylogenetic analysis

Phylogenetic analysis of LipC was performed to evaluate its evolutionary relationship with previously reported lipases. The full-length amino acid sequence of LipC was used as the query to retrieve representative lipase sequences from the NCBI database. Protein sequences were aligned using the ClustalW algorithm.

The phylogenetic tree was constructed using MEGA version 11 based on the neighbor-joining (NJ) method with 1000 bootstrap replicates. Bootstrap values greater than 50% were displayed at the corresponding branch nodes. The evolutionary distances were computed using the Poisson correction method, and the scale bar represents the number of amino acid substitutions per site.

### Gene cloning and construction of recombinant strains

#### Genomic DNA extraction and gene amplification

The wild-type strain *Burkholderia gladioli* Bsp-1 was cultivated in LB medium at 30 °C with shaking at 200 rpm for 24 h. Genomic DNA was extracted using a standard phenol-chloroform method following lysozyme treatment (1 mg/mL, 37 °C, 30 min) and proteinase K digestion.

The lipase gene (*lipC*) and its cognate foldase gene (*lifB*) were amplified based on conserved regions identified from homologous sequences of *Burkholderia gladioli* and closely related *Burkholderia* species available in the NCBI database. Multiple sequence alignment was performed to determine conserved regions suitable for primer design.

Gene-specific primers were designed using Primer Premier 5.0 software. Restriction enzyme recognition sites were incorporated at the 5′ ends of the primers to facilitate directional cloning into expression vectors:

1. *LifB* primers
  - Primer1: atatACATATGGCAGATCTcCTGGCTGGTTCCAGCGCGCC
  - Primer2: gtttcTTTACCAGACTCGAGCTACCCGCCCGCGCCGAGC
2. *LipC* primers
  - Primer1: CAGGATCCGAATTCGGGGCCGGCCACCGTTTCCGA
  - Primer2: GCATTATGCGGCCGCTCAATGCTGCGTTTCCGGCGCCAG

PCR amplification was performed using high-fidelity DNA polymerase. The amplified fragments were purified and sequenced to confirm their identity.

The nucleotide sequences were deposited in GenBank under accession numbers MK590997 (*lipC*) and OQ144967 (*lifB*).

#### Construction of recombinant plasmids and expression strains

The *lipC* gene was inserted into the expression vector pET-22b(+) under the control of the T7 promoter to generate *LipC-pET*. For co-expression, *lipC* was cloned into the first multiple cloning site (MCS1) of pETDuet-1, while *lifB* was inserted into the second multiple cloning site (MCS2). Both genes were driven by independent T7 promoters.

LipC was expressed with a C-terminal His-tag to facilitate purification, whereas LifB was expressed without affinity tags.

Recombinant plasmids were constructed using standard restriction digestion and ligation procedures and verified by colony PCR and DNA sequencing. The confirmed constructs were transformed into *E. coli* Origami 2 (DE3) competent cells. The co-expression strain was designated C-OB, and the strain expressing LipC alone was designated B-OB.

### Culture conditions for producing the lipase

A single colony of the recombinant strain C-OB was inoculated into LB medium and cultivated at 37 ℃ with shaking at 200 rpm until reaching the logarithmic growth phase. The culture was then transferred into auto-induction medium at an inoculation ratio of 1% (v/v) and incubated for 24 h. Cells were harvested by centrifugation at 8000 × g for 10 min at 4 ℃. Auto-induction medium was used to facilitate protein expression without IPTG induction.

### Lipase activity assay

Lipase activity was determined using p-nitrophenyl palmitate (pNPC16) as the substrate. Solution A consisted of 16.5 mM pNPC16 dissolved in isopropanol. Solution B contained 20 mM acetate buffer (pH 4.0) supplemented with 2% (w/v) Triton X-100 and 0.1% (w/v) gum arabic. Solutions A and B were mixed at a ratio of 1:9 (v/v) to prepare the working substrate solution.

The standard reaction mixture contained 450 μL of substrate solution and 50 μL of appropriately diluted enzyme solution. After incubation at 50 ℃ for 10 min, the reaction was terminated by adding 500 μL of 10% (w/v) trichloroacetic acid, followed by 1 mL of 0.5 M Na_2_CO_3_ to adjust the pH to alkaline conditions prior to absorbance measurement at 405 nm. One unit of lipase activity was defined as the amount of enzyme releasing 1 μmol of p-nitrophenol per minute under the assay conditions.

To account for non-enzymatic hydrolysis of pNPC16, especially under acidic conditions, control reactions (blanks) were performed in parallel by replacing the enzyme solution with an equal volume of buffer. The absorbance values of the blanks were subtracted from the corresponding sample readings to obtain the net enzymatic activity.

For determination of pH dependence, the standard assay protocol was followed except that the reaction buffer was replaced with 20 mM acetate buffer (pH 3.0–5.5) for acidic conditions and 20 mM Tris-HCl buffer (pH 7.0–9.0) for neutral to alkaline conditions. Corresponding buffer blanks were included for each pH condition and subtracted to correct for spontaneous substrate hydrolysis (Zhu et al. 2019a).

### Lipase purification

Cell pellets were resuspended in lysis buffer and disrupted by ultrasonication on ice. The cell debris was removed by centrifugation, and the supernatant was subjected to purification.

His-tagged recombinant LipC was purified using immobilized metal affinity chromatography (HisTrap HP, 1 mL; GE Healthcare), followed by desalting using a Sephadex G-25 column (GE Healthcare). The binding buffer contained 20 mM Na_2_HPO_4_/NaH_2_PO_4_ (pH 7.4), 20 mM imidazole, and 500 mM NaCl. Proteins were eluted with the same buffer containing 250 mM imidazole. The purified protein was desalted according to the manufacturer’s instructions and used for subsequent characterization.

### Effect of temperature on activity and stability

The optimal temperature for LipC activity was determined by measuring relative activity at temperatures ranging from 30 to 75 ℃ under standard assay conditions at the optimal pH (pH 4.0, 20 mM acetate buffer).

Thermal stability was evaluated by incubating the enzyme at 25–70 ℃ for 1 h in Na_2_HPO_4_/NaH_2_PO_4_ buffer (pH 6.0). After incubation, residual activity was measured under the standard assay conditions at pH 4.0 (20 mM acetate buffer) and 50 ℃. Enzyme activity without pre-incubation was defined as 100% activity.

### Effect of pH on activity and stability

The optimal pH for LipC activity was determined at 55 ℃ using 20 mM buffer systems covering a pH range of 2.2–10.0. The following buffers were employed: glycine–HCl (pH 2.2–3.0), acetate buffer (pH 3.0–5.5), phosphate buffer (pH 6.0–7.5), Tris–HCl (pH 8.0–9.0), and glycine–NaOH (pH 9.5–10.0).

For each pH condition, control reactions (blanks) were performed by replacing the enzyme solution with the corresponding buffer to account for non-enzymatic hydrolysis of pNPC16. The absorbance of each blank was subtracted from the corresponding sample value.

For pH stability analysis, the enzyme was incubated in the above buffers (20 mM) over a pH range of 2.2–12.0 at 25 ℃ for 24 h. After incubation, residual activity was measured under the standard assay conditions at pH 4.0 (20 mM acetate buffer) and 50 ℃. The untreated enzyme stored at pH 6.0 (20 mM Na_2_HPO_4_/NaH_2_PO_4_ buffer) served as the control (defined as 100% activity).

### Effect of organic solvents on the activity of lipase

The purified enzyme was diluted in 20 mM Na_2_HPO_4_/NaH_2_PO_4_ buffer (pH 6.0), and various organic solvents (n-heptane, n-hexane, cyclohexane, methanol, ethanol, isopropanol, ethyl acetate, acetone, and toluene) were added to a final concentration of 30% (v/v). The mixtures were incubated at 25 ℃ with shaking at 300 rpm for 1 h.

After solvent treatment, aliquots of the incubation mixtures were diluted 20-fold with buffer prior to activity determination, such that the final solvent content during the residual activity assay was ≤ 1.5% (v/v). Residual activity was then measured under the standard assay conditions at pH 4.0 (20 mM acetate buffer) and 50 ℃. For each solvent, a corresponding control sample incubated without solvent was subjected to the same dilution step and defined as 100% activity.

### Effect of metallic ions and protease inhibitor on enzyme activity

To evaluate the effect of metal ions and chemical additives on enzyme stability, the purified LipC was pre-incubated with various metal ions or reagents (β-mercaptoethanol, EDTA, SDS, and DTT) at a final concentration of 10 mM in 20 mM Na_2_HPO_4_/NaH_2_PO_4_ buffer (pH 6.0). The mixtures were incubated at 30 ℃ with shaking at 300 rpm for 1 h.

After incubation, aliquots were diluted 20-fold prior to activity measurement to minimize the concentration of metal ions or additives during the assay (final concentration ≤ 0.5 mM). Residual activity was then determined under standard assay conditions at pH 4.0 (20 mM acetate buffer) and 50 ℃. Enzyme samples incubated under identical conditions without additives were subjected to the same dilution step and defined as 100% activity.

### Substrate specificity and kinetic analysis

Substrate specificity was evaluated using p-nitrophenyl esters with different acyl chain lengths (C2, C4, C8, C10, C12, C14, C16, and C18). Reactions were performed at a substrate concentration of 16.5 mM, pH 4.0, and 50 ℃. Relative activity was calculated by defining the highest activity as 100% (Cao et al. 2012).

For kinetic analysis, substrates ranging from C6 to C18 were used at concentrations of 0.2–5.0 mM. Initial reaction rates were determined from the linear portion of the reaction progress curves. Kinetic parameters (*K*_m_ and *V*_max_) were obtained by non-linear regression fitting to the Michaelis–Menten equation. Catalytic constants (*k*_cat_) and catalytic efficiencies (*k*_cat_/*K*_m_) were calculated based on at least three independent experiments. Lineweaver–Burk plots were generated for visualization.

#### Sequence accession numbers

The nucleotide sequences of *lipC* and *lifB* have been deposited in the GenBank database under accession numbers MK590997 and OQ144967, respectively.

## Results

### Cloning and sequence analysis of lifB and lipC

A lipase-producing wild strain, *Burkholderia gladioli* Bsp-1, was isolated in our laboratory. The lipase gene *lipC* and the cognate foldase gene *lifB* were amplified from genomic DNA using primers designed from conserved regions of homologous genes in closely related *Burkholderia* species. The amplified fragments were sequenced and verified by BLAST analysis. The confirmed sequences were deposited in GenBank under accession numbers MK590997 (*lipC*) and OQ144967 (*lifB*). The whole genome sequence of strain Bsp-1 has not been published.

**Fig. 1:**
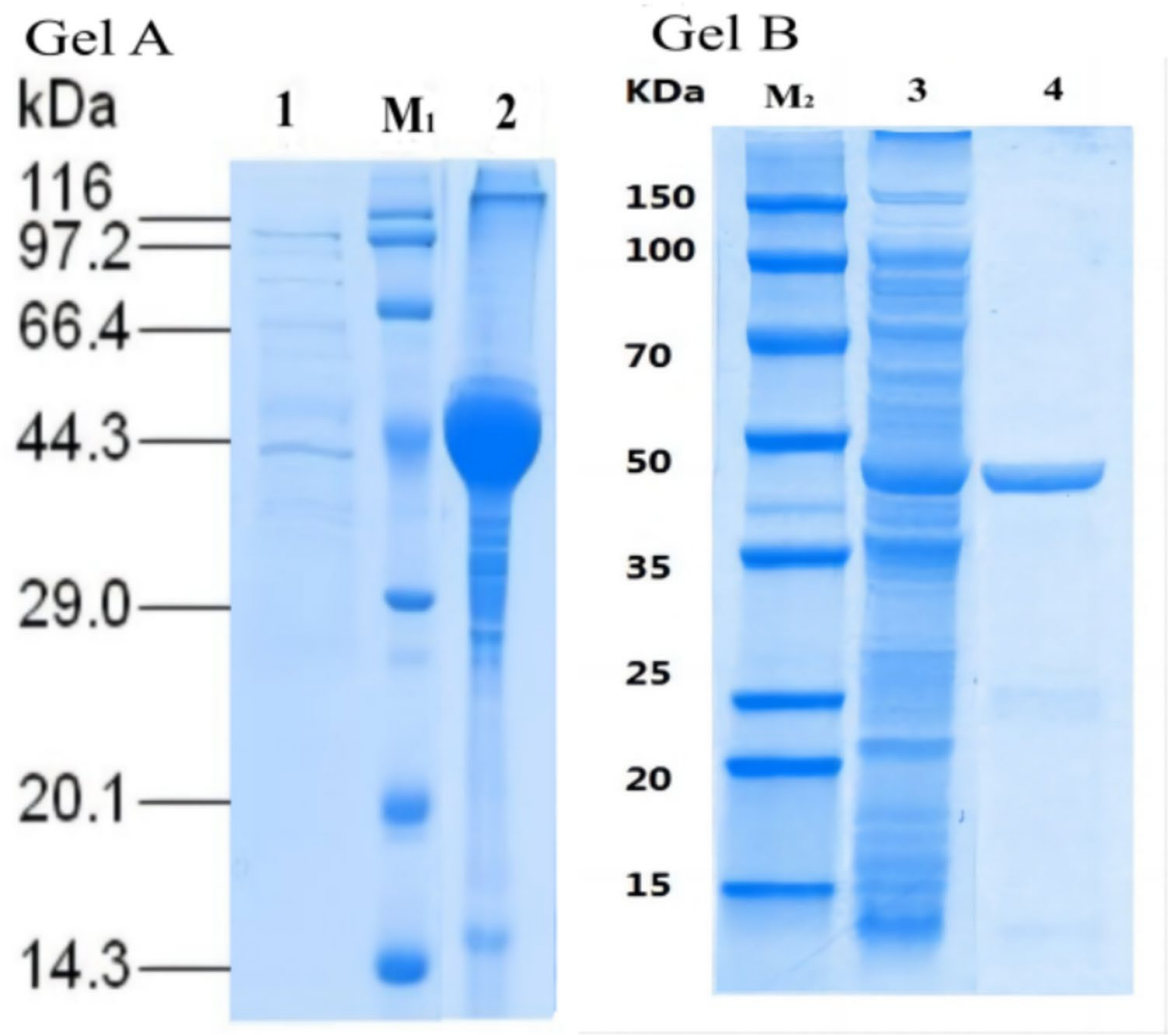
SDS–PAGE analysis of LipC expression with or without foldase LifB. Gel A (LipC alone): Lane M_1_, protein molecular weight marker; Lane 1, soluble fraction of *E. coli* expressing LipC alone; Lane 2, insoluble fraction of *E. coli* expressing LipC alone. Gel B (co-expression): Lane M_2_, protein molecular weight marker; Lane 3, soluble fraction of *E. coli* co-expressing LipC and LifB; Lane 4, purified LipC from co-expression strain (Ni^2+^-affinity)

For heterologous expression, *lipC* was expressed either alone or co-expressed with *lifB* using the pETDuet-1 vector in *Escherichia coli* Origami 2(DE3). As shown in Fig. 1, expression of *lipC* alone resulted predominantly in insoluble protein accumulation, whereas co-expression with the foldase *lifB* resulted in a substantial increase in the soluble fraction of *LipC* and enabled subsequent purification.

BLAST analysis of the deduced amino acid sequence revealed that LipC shared 34.65% sequence identity with *Candida antarctica* lipase A (CALA; PDB ID: 2VEO). Conserved catalytic residues and a putative oxyanion hole characteristic of lipases belonging to the CALA family were identified.

To further evaluate the evolutionary relationship of LipC with previously reported lipases, a phylogenetic analysis was performed based on full-length amino acid sequences. As shown in Fig. S1, LipC clustered within the LipA2 subfamily and formed a distinct branch together with lipases from Burkholderia species, clearly separated from LipA1 and LipA3 subfamilies.

### Expression and purification of LipC

Recombinant LipC was produced by co-expression with the foldase LifB in *E. coli* Origami 2(DE3). The soluble protein fraction was purified using Ni^2^⁺ affinity chromatography followed by desalting. SDS–PAGE analysis showed a predominant single protein band with an apparent molecular mass of approximately 45.6 kDa, consistent with the theoretical molecular weight of LipC (Fig. 1).

Purified LipC obtained via foldase-assisted expression was used for all subsequent biochemical characterization.

### Effect of temperature on enzyme activity and stability

**Fig. 2:**
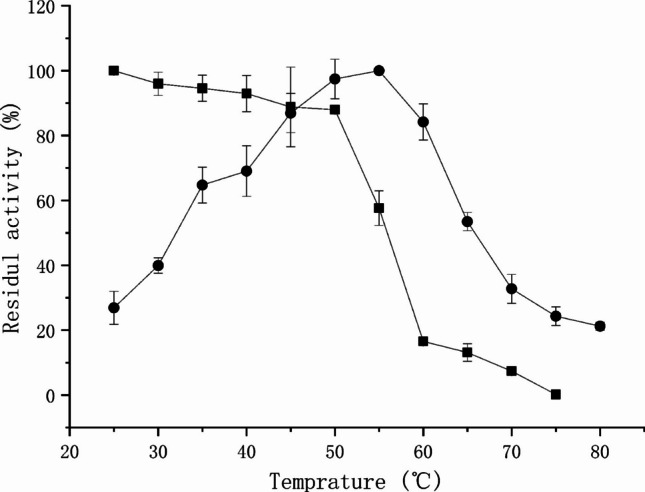
Effects of temperature on the catalytic activity (●) and thermal stability (■) of LipC. For activity determination, reactions were carried out at the indicated temperatures under standard assay conditions. For thermal stability analysis, the enzyme was pre-incubated at the indicated temperatures for 1 h, and residual activity was subsequently measured under standard assay conditions

The effect of temperature on LipC activity and stability is shown in Fig. 2. LipC exhibited maximal activity at 55 ℃. The enzyme retained relatively high stability between 25 and 55 ℃, maintaining approximately 60% of its initial activity after incubation at 55 ℃ for 1 h. In contrast, enzyme activity decreased sharply at temperatures above 60 ℃, with residual activity dropping to approximately 20% after incubation at 60 ℃ for 1 h.

### Effect of pH on enzyme activity and stability

**Fig. 3:**
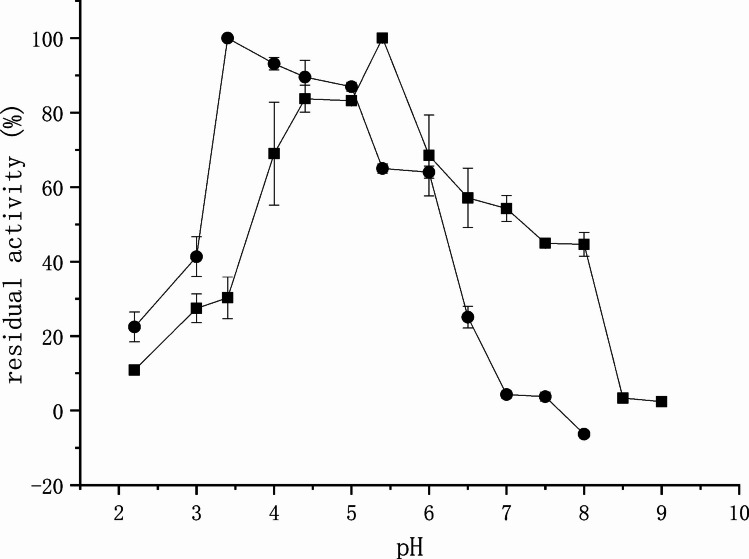
Effects of pH on the catalytic activity (●) and pH stability (■) of LipC. For activity determination, reactions were performed at 50 ℃ in the indicated buffers. For stability analysis, the enzyme was pre-incubated at the indicated pH for 24 h at 25 ℃, and residual activity was subsequently measured under standard assay conditions

The influence of pH on LipC activity and stability is presented in Fig. 3. LipC displayed optimal activity at pH 3.5. However, its stability at this pH was limited, with approximately 30% residual activity after 24 h incubation. In contrast, the enzyme exhibited significantly higher stability in the pH range of 4.0–6.0, retaining more than 70% of its initial activity after incubation at pH 4.0 for 24 h.

### Effect of organic solvents on enzyme activity

**Fig. 4:**
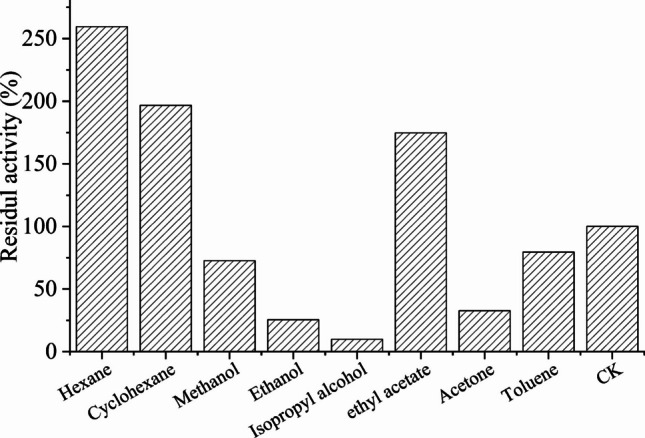
Effect of organic solvents on the residual activity of LipC

The effects of various organic solvents on LipC activity were evaluated, and the results are summarized in Fig. 4. Non-polar solvents with low polarity indices, including n-hexane and cyclohexane, markedly enhanced LipC activity. After incubation in 30% (v/v) n-hexane and cyclohexane for 1 h, relative activities reached approximately 280% and 200% of the control, respectively.

Moderate activation was also observed in the presence of ethyl acetate, with enzyme activity reaching approximately 180% of the control. In contrast, polar solvents such as methanol, ethanol, and isopropanol partially inhibited LipC activity.

### Effect of metal ions and inhibitors on enzyme activity

**Fig. 5:**
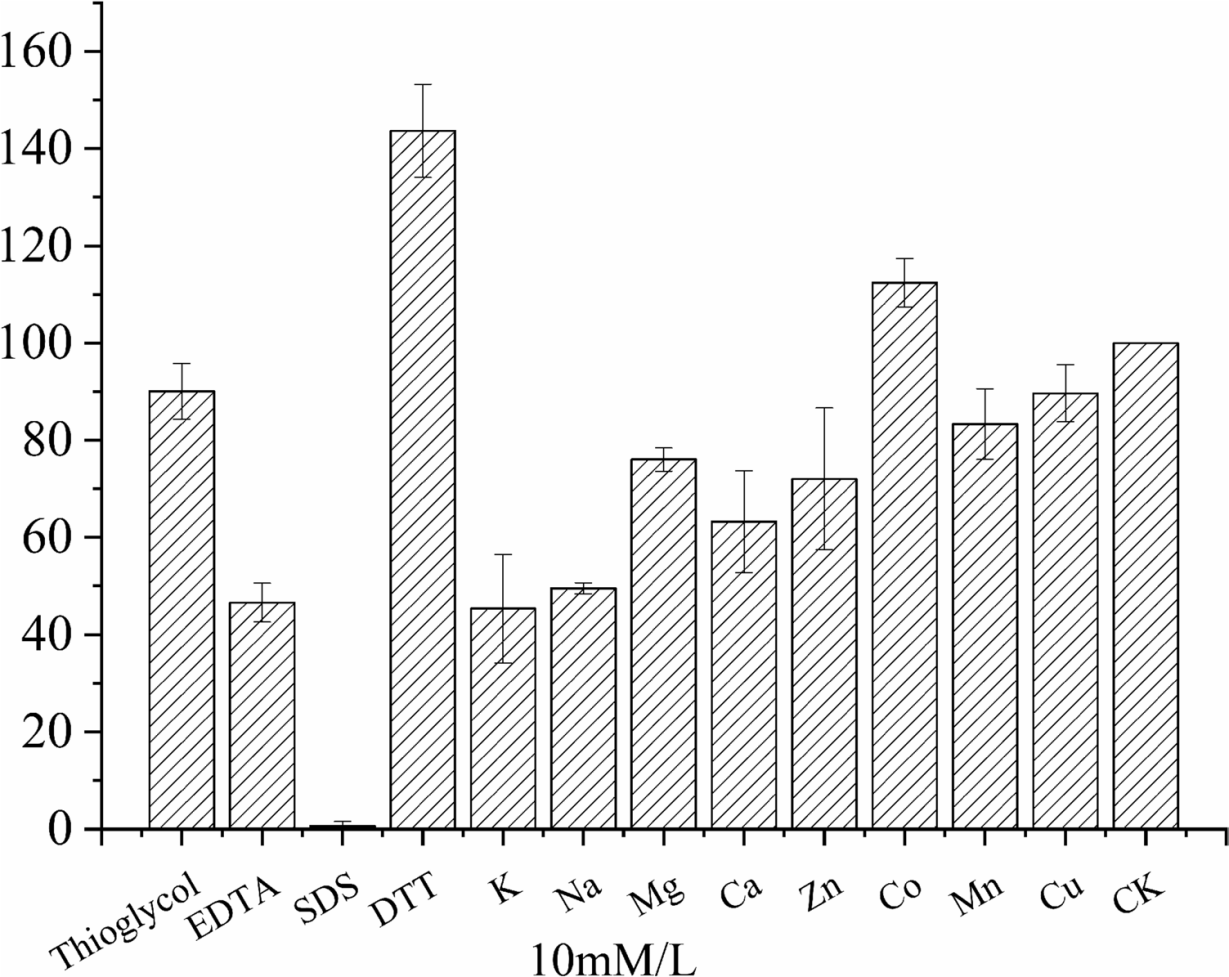
Effects of metallic ions and protein inhibitors on the activity of lipase LipC

The effects of metal ions and inhibitors on LipC activity are shown in Fig. 5. Incubation with Ca^2^⁺ (10 mM) reduced enzyme activity to approximately 70% of the control. The metal chelator EDTA (10 mM) also significantly inhibited LipC activity.

β-mercaptoethanol had no noticeable effect on enzyme activity, whereas SDS strongly inhibited LipC activity. In contrast, DTT enhanced enzyme activity, with residual activity increasing to approximately 150% of the control after incubation for 1 h.

### Substrate specificity and kinetic analysis

**Fig. 6:**
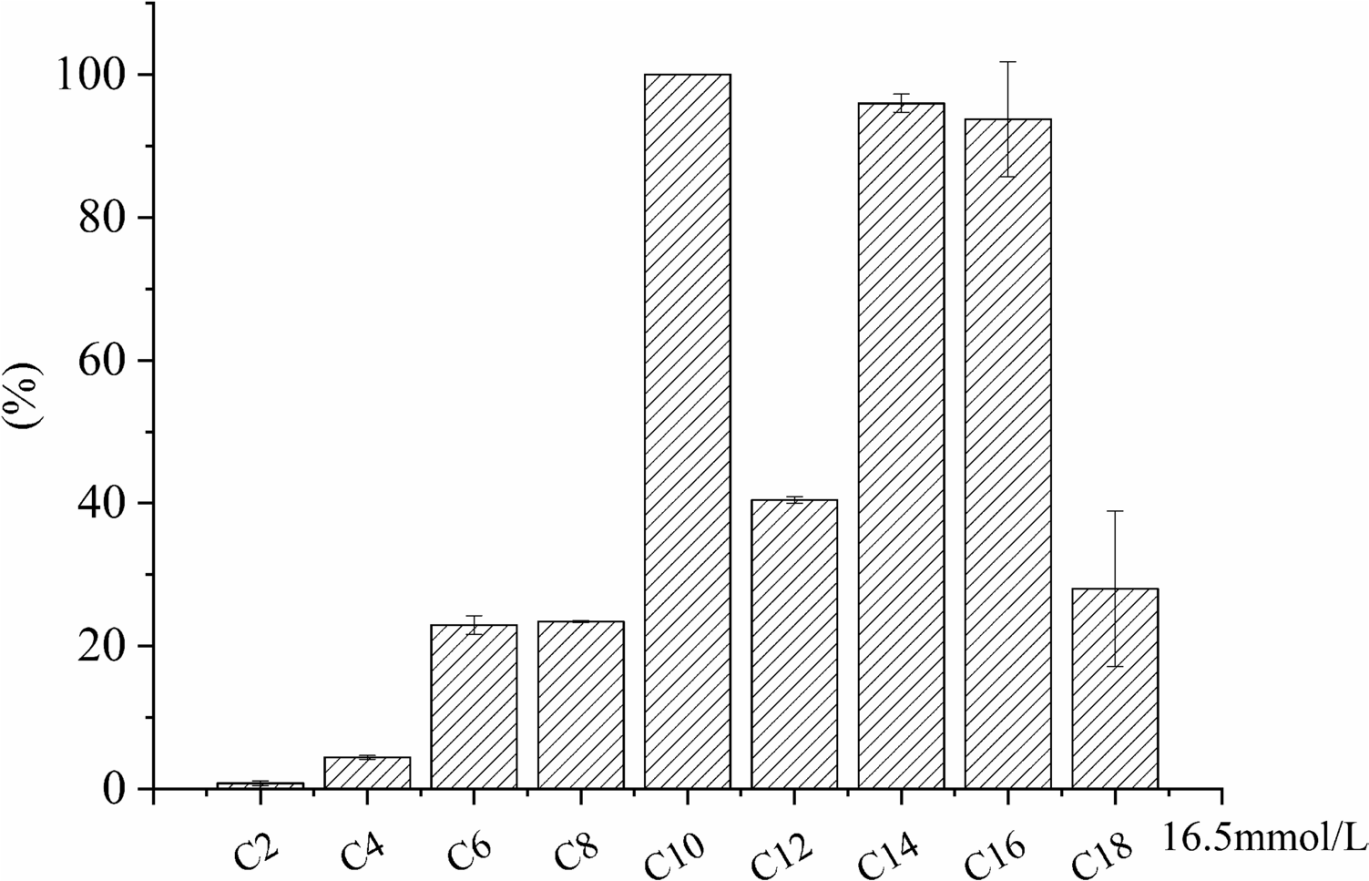
Substrate specificity of lipase LipC

Substrate specificity analysis revealed that LipC displayed minimal activity toward short-chain esters (C2–C8). Maximal catalytic activity was observed for p-nitrophenyl caprate (C10), with comparatively high activity toward C14 and C16. Notably, activity toward C12 and C18 was substantially lower (below 40% of the maximum), suggesting that LipC exhibits a defined chain-length preference rather than a broad specificity toward all medium- and long-chain substrates (Fig. 6).

Kinetic parameters of LipC toward substrates ranging from C6 to C18 were determined and are summarized in Table 1. Among the tested substrates, LipC showed the highest catalytic efficiency toward p-nitrophenyl caprate (C10). The maximum specific activity toward C10 reached 4208.39 U mg^−1^ under the assay conditions. The corresponding kinetic parameters were *K*_m_ = 9.10 mM, *k*_cat_ = 126.30 s^−1^, and *k*_cat_*/K*_m_ = 13.88 s^−1^ mM^−1^.

**Table 1:** Kinetic parameters of lipase LipC for p-nitrophenyl esters

Substrate	*V*_max_ (µmol min^−1^ mg^−1^)	*K*_m_ (mM)	*k*_cat_ (s^−1^)	*k*_cat_/*K*_m_ (s^−1^ mM^−1^)
C6	71.21	9.48	43.58	4.55
C8	45.50	4.11	27.58	6.71
C10	208.39	9.10	126.30	13.88
C12	44.41	3.37	26.92	7.99
C14	119.14	6.78	72.21	10.65
C16	97.56	4.92	59.13	12.02
C18	74.33	6.38	45.05	7.06

## Discussion

### Foldase-dependent maturation of LipC

Functional expression of gram-negative bacterial lipases in *Escherichia coli* frequently requires the assistance of a cognate lipase-specific foldase (Lif/LipB/Lim), which acts as a steric chaperone to promote productive folding and activation of the lipase. In the absence of such foldases, many recombinant lipases accumulate as insoluble inclusion bodies or remain catalytically inactive, a phenomenon that has been widely reported for lipases from *Pseudomonas* and *Burkholderia* species (Madan et al. 2010; Peng et al. 2011; Putra et al. 2019; Shu et al. 2019).

In the present study, LipC expressed alone in *E. coli* was recovered predominantly in the insoluble fraction, whereas co-expression with its cognate foldase LifB resulted in a substantial increase in the soluble fraction and enabled purification of catalytically active LipC. This clear dependence on LifB demonstrates that LipC follows the canonical foldase-assisted folding pathway characteristic of many LipA-type bacterial lipases. Similar foldase-dependent behavior has been documented for lipases from *Burkholderia* *cepacia* and *Pseudomonas aeruginosa*, where co-expression of lipase and foldase converts inactive or insoluble recombinant protein into a soluble and functional enzyme (Madan et al. 2010; Peng et al. 2011; Shu et al. 2019).

Although foldase dependence is widely regarded as a defining feature of most I.1/I.2 lipases, emerging evidence indicates that it is not universally conserved across this group. Several family I.1 lipases, including metagenome-derived representatives and specific phylogenetic sublineages, have been reported to achieve active conformations in the absence of a dedicated foldase (Pauwels 2008; Viegas et al. 2020). In addition, single amino acid substitutions in otherwise canonical LipA enzymes have been shown to partially alleviate foldase dependence, suggesting that the requirement for a foldase may reflect the presence of specific folding-energy barriers rather than an absolute structural necessity (Pauwels et al. 2005). Therefore, foldase dependence appears to be clade- or structure-specific and cannot be reliably inferred solely from LipA-like sequence annotation.

Taken together, our results provide direct experimental evidence that LipC belongs to the foldase-dependent subgroup of LipA-type lipases. This characteristic has important practical implications for heterologous expression and biotechnological production, as efficient preparation of active LipC requires coordinated expression of both the lipase and its cognate foldase.

### Phylogenetic placement of LipC

Phylogenetic analysis based on full-length amino acid sequences placed LipC within the LipA-type family of gram-negative bacterial lipases, clustering with homologs from *Burkholderia* species in the LipA2-associated group (Fig. S1). This positioning is consistent with conserved sequence motifs and catalytic residues typical of proteobacterial LipA-type lipases, which form a major lineage within the broader class of α/β-hydrolase lipases (Arpigny and Jaeger 1999; Kim et al. 2008).

Notably, LipC did not group with filamentous fungal acid lipases, which represent a phylogenetically distinct lineage despite sharing the canonical α/β-hydrolase fold. This indicates that the pronounced acidophilicity of LipC does not result from evolutionary proximity to fungal acid lipases, but instead has emerged within a bacterial LipA-type framework. Such decoupling of phylogenetic origin and pH preference, although uncommon, has been reported for certain bacterial lipases (Kovacic et al. 2019; Ryu et al. 2006).

Comparison of LipC with its closest non-acidophilic homolog within the LipA2-associated cluster further provides insight into the potential structural basis of its acidophilic behavior. Sequence alignment revealed that the catalytic triad (Ser–Asp–His) and conserved α/β-hydrolase core motifs are fully preserved, consistent with the conserved structural framework of LipA-type lipases (Ohara et al. 2014), indicating that the fundamental catalytic architecture remains unchanged. However, several notable differences were observed at the level of surface-exposed residues and predicted physicochemical properties.

First, LipC displays a moderately increased proportion of acidic residues (Asp and Glu) relative to its closest non-acidophilic homolog, accompanied by a slight reduction in basic residues (Lys and Arg). Such shifts in surface charge composition are frequently associated with enhanced structural stability under acidic conditions (Pham et al. 2018), as enrichment of negatively charged residues may reduce electrostatic repulsion and favor intramolecular stabilization in proton-rich environments. Acid-stable enzymes are frequently characterized by increased negative surface potential and reduced pH-sensitive electrostatic repulsion (Beliën et al. 2009).

Second, theoretical isoelectric point (pI) analysis indicates that LipC possesses a lower predicted pI compared to the closest non-acidophilic counterpart, consistent with its functional preference for acidic pH. Lower pI values have been correlated with acid adaptation in various enzymes, reflecting altered surface electrostatics and protonation equilibria. Third, inspection of sequence alignment suggests localized variations in loop regions adjacent to the predicted lid domain and acyl-binding entrance. Even subtle modifications in these flexible regions can influence substrate accessibility, conformational dynamics, and pH-dependent stability without altering the conserved catalytic core. Such adaptive surface-level modifications are consistent with previous reports showing that environmental pH preference can evolve through limited but strategically positioned residue substitutions (Cockburn et al. 2011). Collectively, these observations support the notion that the acidophilic character of LipC does not arise from a novel fold or catalytic mechanism, but rather from incremental sequence-level adaptations superimposed on a conserved LipA-type structural scaffold. Further structural modeling or electrostatic surface analysis would be required to precisely delineate the contribution of these features.

The placement of LipC within the LipA2-type cluster therefore indicates that its distinctive biochemical properties, including acid tolerance and solvent stability, expand the functional diversity of an otherwise well-characterized lipase lineage. This observation reinforces the concept that substantial biochemical divergence can arise within a conserved phylogenetic scaffold through relatively limited but strategically positioned sequence changes.

### Acidophilic behavior and stability of LipC

Acidophilic lipases are most commonly reported from filamentous fungi and yeasts, where enzymes from *Aspergillus* and *Candida* species typically exhibit pH optima between 3.0 and 4.0 and retain stability under acidic conditions (Nur et al. 2023; Shi et al. 2014; Xing et al. 2021). In contrast, most bacterial lipases described to date display neutral or alkaline pH optima. Nevertheless, several acid-active bacterial lipases have been reported. For example, an acidic Lip I.3 from a *Pseudomonas fluorescens*–like strain exhibits unusual catalytic properties under acidic conditions (Panizza et al. 2013), and an acidic lipase from *Pseudomonas gessardii* has been characterized and applied in immobilized systems (Kandasamy et al. 2010). In addition, extremely acidic lipases have been described from Bacillus species (Saranya et al. 2014). These reports demonstrate that bacterial acidophilic lipases, although less common than fungal counterparts, are not unprecedented.

Against this background, LipC exhibits maximal activity at pH 3.5 and retains more than 70% of its activity after prolonged incubation at pH 4.0, positioning it among the more strongly acid-tolerant bacterial lipases reported to date. Notably, several previously described bacterial acid lipases display optimal activities closer to pH 4.5–5.0, whereas LipC operates efficiently at lower pH values. Thus, the acidophilicity of LipC approaches that typically observed in fungal lipases while maintaining the structural framework of a gram-negative LipA-type enzyme.

In terms of temperature stability, LipC exhibited maximal activity at 55 °C and retained substantial activity after incubation at elevated temperatures, consistent with the stability range commonly reported for many non-thermophilic gram-negative bacterial lipases (Shi et al. 2014; Xing et al. 2021). While LipC does not reach the extreme thermostability of engineered or extremophilic lipases, its combination of moderate thermal robustness and pronounced acid tolerance distinguishes it from many previously characterized bacterial lipases and expands the known functional diversity within this group.

Together, these properties suggest that LipC represents a bacterial LipA-type enzyme that has undergone adaptive modification enabling efficient catalysis under acidic conditions without sacrificing structural stability, thereby broadening the biotechnological potential of bacterial acidophilic lipases.

### Solvent tolerance of LipC

The ability of lipases to retain activity in organic solvents is a key property for nonaqueous biocatalysis, where hydrophobic media can enhance substrate solubility and shift reaction equilibria toward synthesis rather than hydrolysis (Koskinen and Klibanov 1996; Salihu and Alam 2015). In such systems, enzyme performance depends largely on the preservation of a minimal hydration shell and conformational integrity in low-polarity environments, which is typically favored in nonpolar solvents such as n-hexane and cyclohexane (Gagnon 2013; Hirata et al. 1990).

In this study, LipC showed pronounced activation in nonpolar solvents, particularly n-hexane and cyclohexane, where relative activities exceeded those measured in aqueous buffer. Similar solvent-induced activation has been reported for several microbial lipases and is commonly attributed to favorable solvent–enzyme interactions that stabilize catalytically competent conformations or promote more efficient substrate access to the active site (Kajiwara et al. 2017; Kim et al. 1984). In contrast, polar solvents such as methanol, ethanol, and isopropanol partially inhibited LipC activity, consistent with their tendency to strip essential bound water molecules and destabilize protein structure (Hirata et al. 1990; Sharma and Kanwar 2014).

The solvent tolerance of LipC therefore places it within the group of naturally solvent-tolerant lipases that function efficiently in hydrophobic media without immobilization or extensive protein engineering. This behavior has been observed for selected bacterial and fungal lipases, including solvent-stable *Pseudomonas* and *Candida* enzymes, which are widely used for esterification and transesterification reactions in organic solvents (Cabanding et al. 2025; Dhake et al. 2012; Mathpati and Bhanage 2016). The strong activity of LipC in n-hexane and cyclohexane, together with its acidophilic nature, highlights a combination of traits that is rarely reported for bacterial lipases and is particularly attractive for biocatalytic processes operating under acidic and low-water conditions.

### Metal discussion

The observed inhibitory effects of both Ca^2+^ and EDTA suggest that LipC is unlikely to function as a classical metal-dependent enzyme. In several bacterial lipases, Ca^2+^ plays a structural role by stabilizing the active-site conformation or enhancing thermal stability. However, excessive Ca^2+^ concentrations may also induce conformational perturbations or alter surface charge distribution, leading to reduced activity.

The inhibition observed in the presence of EDTA does not necessarily indicate strict metal dependence but may reflect indirect structural effects, as chelating agents can disturb weakly bound structural metal ions or interfere with protein folding. Similar behavior has been reported for certain Burkholderia lipases belonging to family I.2, which are primarily foldase-dependent rather than metal-dependent enzymes.

Therefore, the metal ion sensitivity profile of LipC appears to be consistent with previously characterized foldase-dependent bacterial lipases.

### Substrate specificity and catalytic efficiency of LipC

Substrate chain-length preference is a defining feature that distinguishes lipases from esterases, with true lipases preferentially hydrolyzing medium- to long-chain fatty acid esters rather than short-chain substrates (Wang et al. 2025). In agreement with this criterion, LipC displayed low activity toward short-chain esters (C2–C8) but markedly higher activity toward medium- and long-chain substrates (C10–C16), confirming its classification as a bona fide lipase rather than an esterase.

Among the tested substrates, LipC showed the highest catalytic efficiency toward p-nitrophenyl caprate (C10), which is a chain-length preference commonly reported for many microbial lipases. Several fungal and bacterial lipases, including enzymes from *Candida*, *Geotrichum*, and *Rhizopus*, exhibit maximal or near-maximal activity toward C8–C10 substrates, reflecting binding-pocket geometries that favor medium-chain acyl groups (Ding et al. 2019; Holmquist et al. 1997; Jallouli et al. 2015).

An intriguing observation in the substrate specificity profile is the markedly reduced activity toward the C12 substrate compared with C10, C14, and C16 esters. This discontinuity indicates that the chain-length preference of LipC does not follow a simple monotonic trend but instead reflects discrete structural constraints within the acyl-binding channel (Schmitt et al. 2002). The pronounced decrease in activity toward C12 is particularly noteworthy, as it lies between highly active C10 and C14 substrates. Such a pattern suggests the presence of a defined accommodation window within the hydrophobic tunnel rather than a gradual chain-length effect.

In α/β-hydrolase lipases, substrate discrimination is dictated by the depth, contour, and hydrophobic character of the acyl-binding pocket, as well as lid-domain positioning (Alejaldre et al. 2022; Miguel-Ruano et al. 2021). It is plausible that the C12 acyl chain neither fully occupies the distal hydrophobic region accessible to longer substrates (C14–C16) nor achieves the optimal packing configuration observed for C10 (Santarossa et al. 2005). This suboptimal positioning may impair precise alignment of the scissile ester bond with the catalytic serine, thereby reducing catalytic efficiency. Similar chain-length discontinuities have been reported in other bacterial lipases and are generally attributed to subtle steric mismatches or tunnel-geometry constraints rather than fundamental changes in catalytic machinery (Pinotsis et al. 2023).

Although detailed structural or docking analyses would be required to definitively resolve this phenomenon, the current data strongly suggest that the substrate-binding pocket of LipC is optimally configured for acyl chains slightly shorter (C10) or longer (C14–C16) than C12 (Santarossa et al. 2005).

The kinetic parameters of LipC further support this conclusion. The* k*_cat_/*K*_m_  values obtained for medium-chain substrates fall within the range reported for characterized microbial lipases and are significantly higher than those typically observed for esterases acting on short-chain substrates. Such enhanced catalytic efficiency toward medium-chain esters is generally attributed to favorable accommodation of the acyl chain within the hydrophobic substrate-binding pocket, leading to both reduced *K*_m_ and increased turnover rates (Sayari et al. 2007; Yang et al. 2002). Thus, the kinetic profile of LipC aligns with that of established medium-chain–preferring lipases.

Taken together, the substrate specificity and catalytic efficiency of LipC, characterized by a clear preference for C10 substrates and favorable kinetic parameters toward medium-chain esters, place this enzyme within the functional spectrum of true lipases. When combined with its foldase dependence, acidophilic behavior, and solvent tolerance, LipC represents a bacterial lipase with a distinctive and industrially relevant set of biochemical properties.

## Conclusion

This study reports the heterologous expression and biochemical characterization of a LipA-type bacterial lipase, LipC, from *Burkholderia gladioli* functional production of LipC in *Escherichia coli* strictly required co-expression with its cognate foldase LifB, highlighting the importance of foldase-assisted folding for this enzyme. LipC exhibited pronounced acidophilic behavior, with optimal activity under acidic conditions, and retained substantial activity in nonpolar organic solvents. These properties distinguish LipC from most of the previously characterized bacterial lipases, which typically display neutral or alkaline pH optima and limited solvent tolerance. Together, the foldase dependence, acid stability, and solvent tolerance of LipC expand the functional diversity of bacterial lipases and provide a reference for future studies exploring bacterial enzymes operating under acidic and low-water conditions.

## Supplementary Information

Below is the link to the electronic supplementary material.

ESM 1 (PDF 195 KB)

ESM 2 (DOCX 144 KB)

## Data Availability

The nucleotide sequences of lipC and lifB have been deposited in the GenBank database under accession numbers MK590997 and OQ144967. Other datasets generated and/or analyzed during the current study are available from the corresponding author on reasonable request.
