# Supplementary material for: Co-expression, purification, and characterization of an acidophilic and n-hexane-tolerant lipase with its foldase from Burkholderia gladioli Bsp-1
Source: Appl Microbiol Biotechnol. 2026 Mar 28;110:128. doi: 10.1007/s00253-026-13788-z (PMC13035632; doi:10.1007/s00253-026-13788-z)
Supplement: Supplementary file 1 [file 253_2026_13788_MOESM1_ESM.pdf]

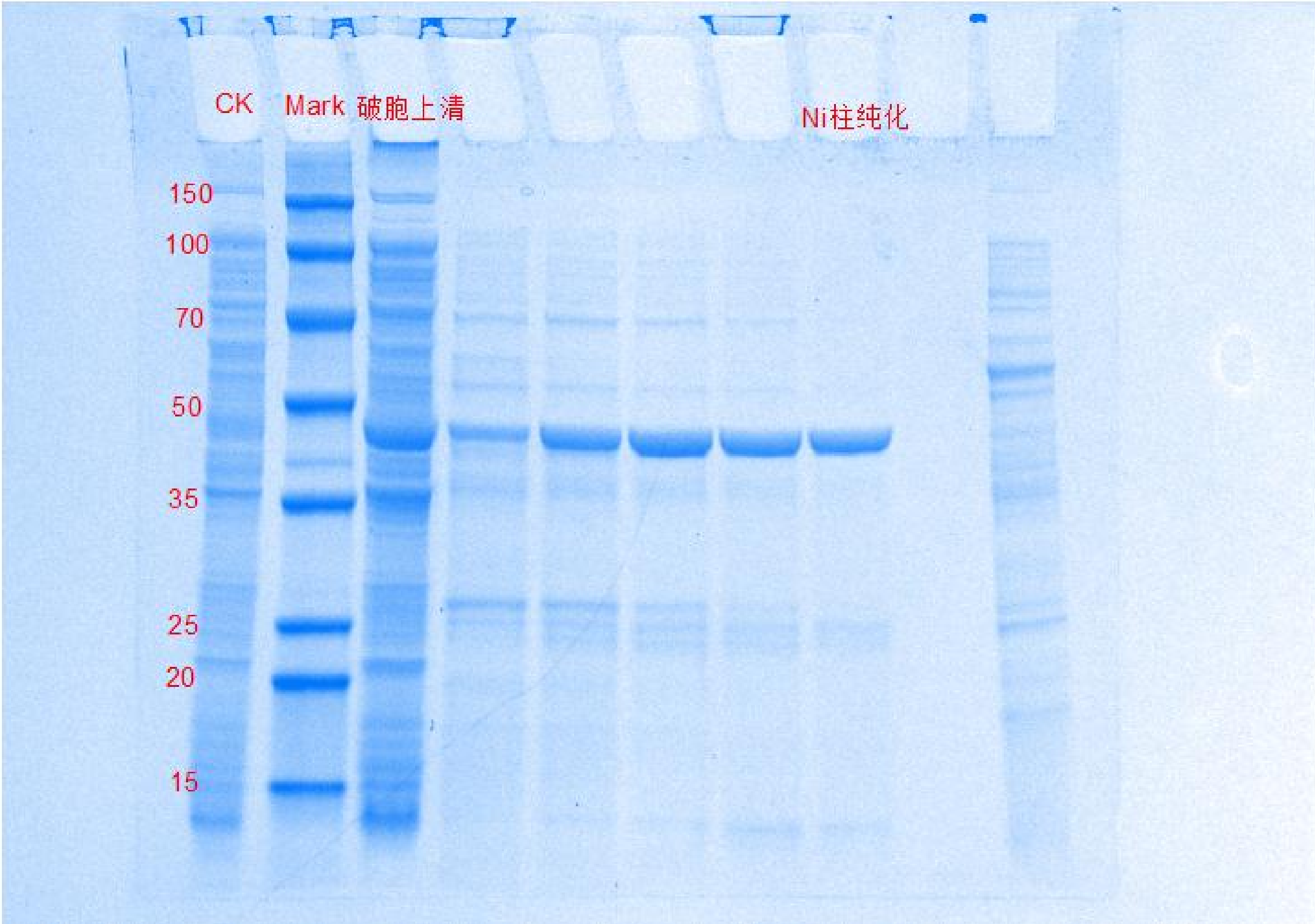

Supplementary Fig. S1

Full uncropped SDS–PAGE gel for LipC co-expressed with LifB (corresponding to Fig.1, lanes 1–2).

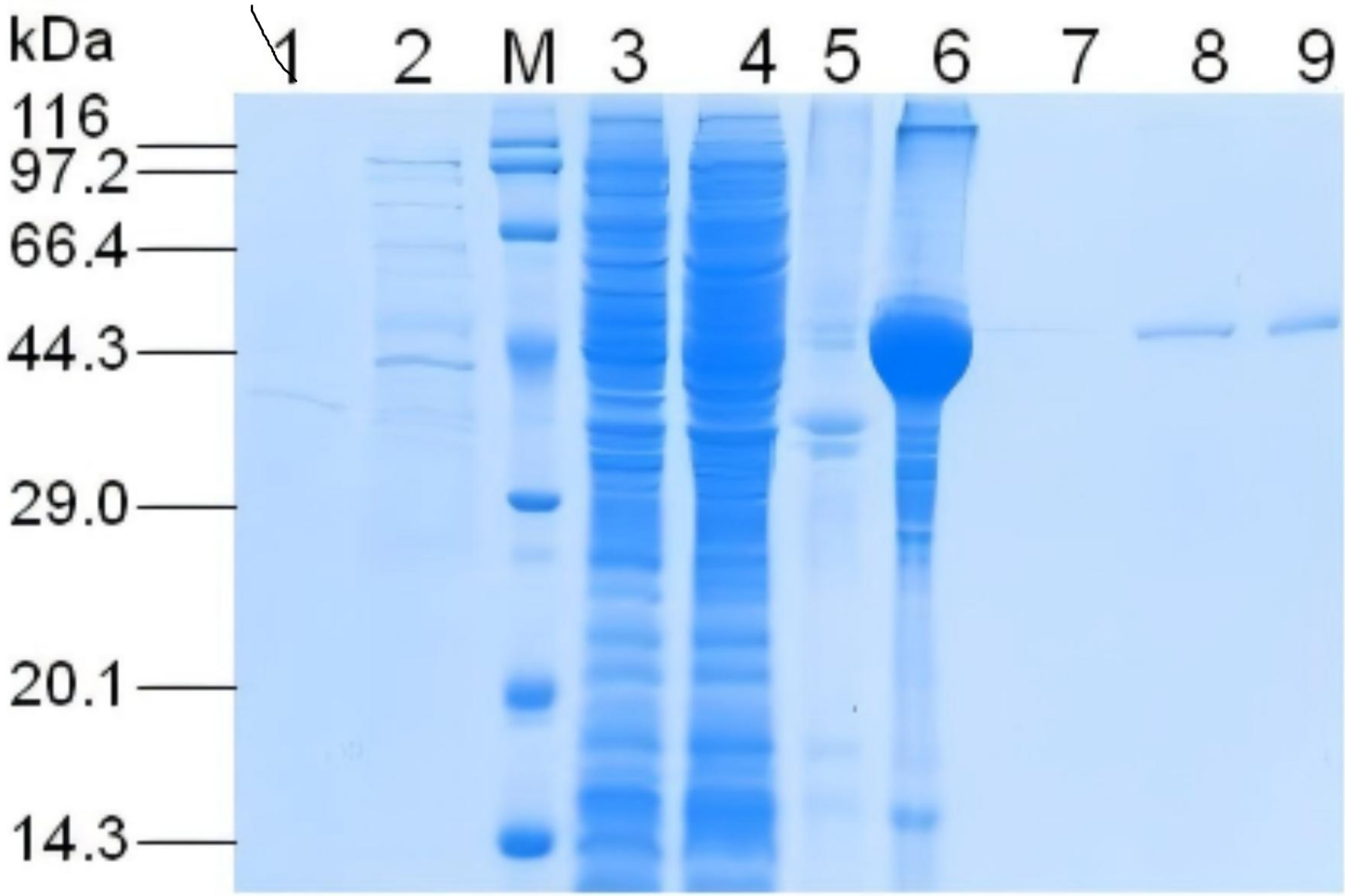

Supplementary Fig. S2

Statement on availability of original gel images

The original uncropped gel image corresponding to the LipC expressed without LifB in , lanes 3– 4 is no longer available because the experiment was performed prior to the current data archiving policy and only the processed image used for figure preparation was retained. The authors confirm that the processed image accurately reflects the experimental result and that no manipulation affecting scientific interpretation was performed.
