# Supplementary material for: Co-expression, purification, and characterization of an acidophilic and n-hexane-tolerant lipase with its foldase from Burkholderia gladioli Bsp-1
Source: Appl Microbiol Biotechnol. 2026 Mar 28;110:128. doi: 10.1007/s00253-026-13788-z (PMC13035632; doi:10.1007/s00253-026-13788-z)
Supplement: Supplementary file 2 [file 253_2026_13788_MOESM2_ESM.docx]

Figure S1

Phylogenetic analysis of LipC and related bacterial lipases based on amino acid sequences.
